# Supplementary material for: Combined Inhibition of Indolamine-2,3-Dioxygenase 1 and C-X-C Chemokine Receptor Type 2 Exerts Antitumor Effects in a Preclinical Model of Cervical Cancer
Source: Biomedicines. 2023 Aug 16;11(8):2280. doi: 10.3390/biomedicines11082280 (PMC10452145; doi:10.3390/biomedicines11082280)
Supplement: Supplementary file 1 [file biomedicines-11-02280-s001.zip › Figure S1.pdf]

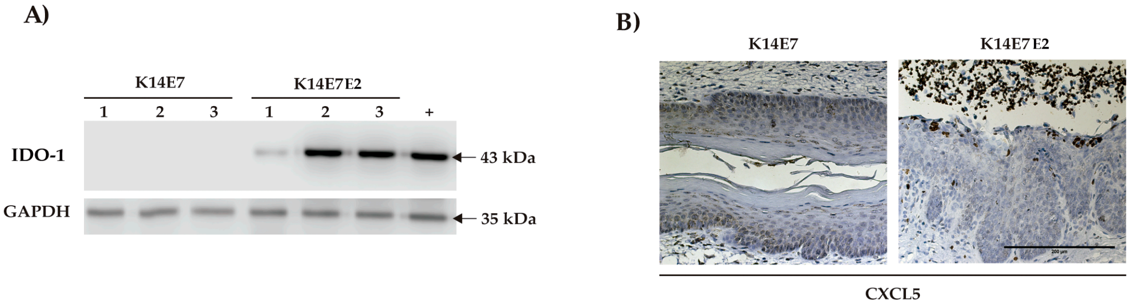

Figure S1. Expression of IDO-1 and CXCL5. A) Detection of IDO-1 by WB in a cervical cancer mouse model (K14E7E2) compared to control (K14E7). B) Immunodetection pattern of CXCL5 in cervical tissue from K14E7E2 compared to K14E7. The visual field at 20x magnification and scale bar 200 μm.
