# Supplementary material for: Combined Inhibition of Indolamine-2,3-Dioxygenase 1 and C-X-C Chemokine Receptor Type 2 Exerts Antitumor Effects in a Preclinical Model of Cervical Cancer
Source: Biomedicines. 2023 Aug 16;11(8):2280. doi: 10.3390/biomedicines11082280 (PMC10452145; doi:10.3390/biomedicines11082280)
Supplement: Supplementary file 1 [file biomedicines-11-02280-s001.zip › Figure S2.pdf]

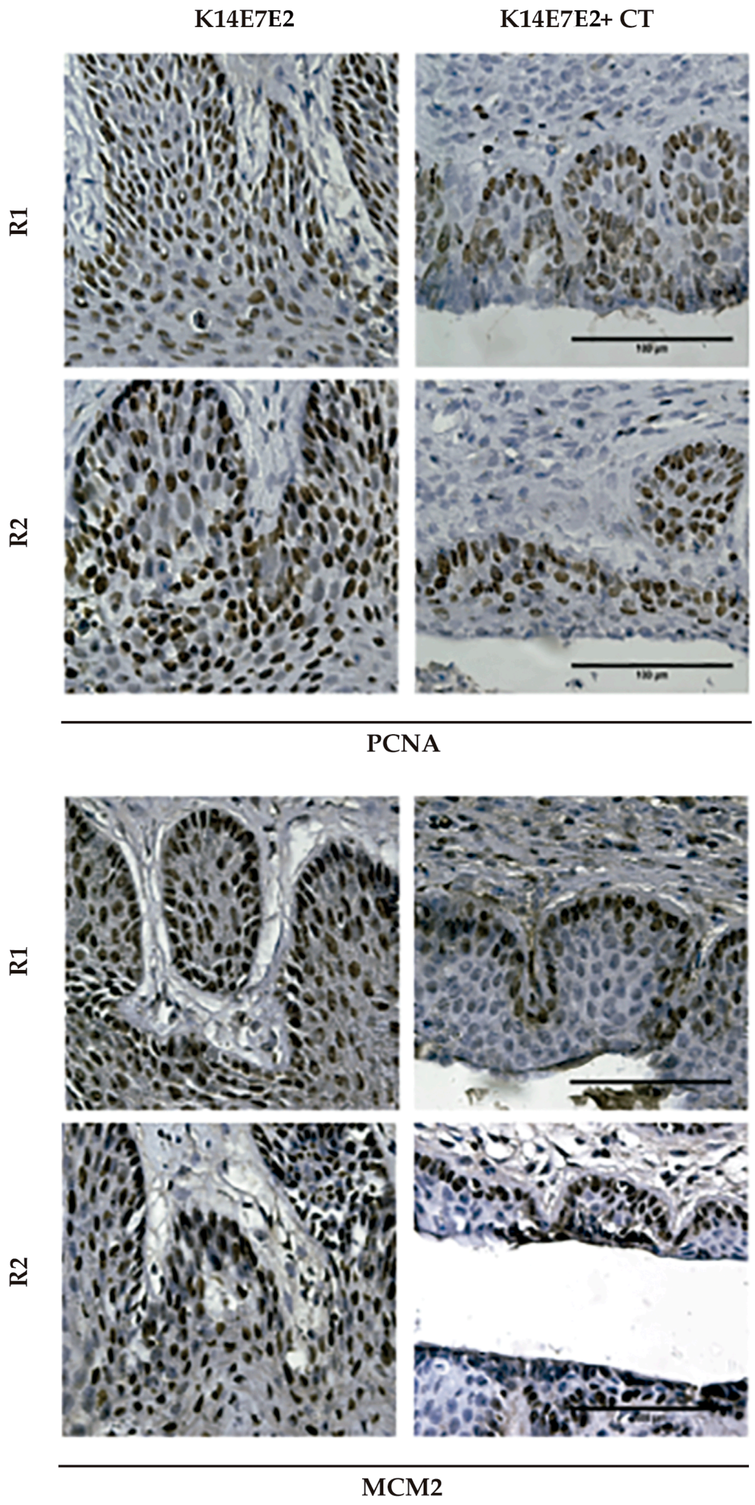

Figure S2. The immunodetection pattern of PCNA A) and MCM2 B) shows a decrease in proliferation when combined therapy is administered (panel K14E7E2 + CT). The visual field at 40x magnification and scale bar 200 μm.
